# Editorial: Scaffold Technology, Tissue and Organ Engineering: New Horizons in Surgery

**DOI:** 10.3389/fsurg.2022.914080

**Published:** 2022-05-18

**Authors:** Lorenzo Cobianchi, Andrea Peloso

**Affiliations:** ^1^ Department of Clinical, Diagnostic and Pediatric Sciences, University of Pavia, Pavia, Italy; ^2^ Department of General Surgery, IRCCS Policlinico San Matteo Foundation, Pavia, Italy; ^3^ Department of Visceral and Transplantation Surgery, University of Geneva Hospitals, Geneva, Switzerland

**Keywords:** scaffold, organ regeneration, surgery, stem cells, tissue engineering

**Editorial on the Research Topic**

***Scaffold Technology, Tissue and Organ Engineering: New Horizons in Surgery***

Regenerative medicine is a vast and highly topical theme with many important aspects to consider. What appears relevant is that the ability to regenerate a function of cells and tissues has never actually been exploited until today. Key topics are represented by, but not limited to, revolutionary advances in the use of acellular dermal matrices (1), treatments with stem cells (2), 3D printers (3) and bioscaffolds (4).

Regenerative medicine represents the therapeutic solution of the next future, allowing patients, whose needs are unmet today, to find a feasible cure, introducing new, more effective treatments into clinical practice. Regenerative medicine looks promising not only from a purely clinical perspective (5). For instance, it will make it possible to offer a sound alternative in the treatment of chronic diseases affecting an ageing population, which represents today an increasing expense for the health system and society. Therefore, the development of regenerative medicine will expect to disrupt the organizational side of clinical institutions, the role and tasks of medical doctors, the treatment options for patients, and the inputs coming from medical and healthcare education.

A fundamental prerogative of regenerative medicine is its multidisciplinarity. This characteristic implies that, in order to rapidly optimize marketable products with a strong innovative character, effective coordinated cooperation must be undertaken among different stakeholders, including industry actors and public research bodies, capable of bringing together the various technologies and knowledge. The researchers and industry experts involved in this investigation (medical doctors, biotechnologists, chemists, physicists, geneticists, biologists, bioengineers, to name a few) have the primary need to “speak the same language,” allowing them to translate their technical knowledge for organic and integrated research (6).

All surgical disciplines will benefit from advances in regenerative medicine (7). Therefore, surgeons should become acquainted with these emerging technology. Although the complete clinical translation still appears challenging, the path taken by research in the field of tissue engineering has as its point of arrival in organ replacement, which represents the quintessence of the cure of every pathology.

In this complex and evolving research scenario, several research groups all over the globe are concentrating their efforts on the progress of regenerative medicine, step by step towards its clinical translation. The aim of the special topic call “Scaffold Technology, Tissue and Organ Engineering: New horizons in surgery” was to gather the most recent experiences to understand the current state-of-the-art and to make an accurate forecast about the future of this type of medical application.

The research topic gathered four contributions: two reviews, one original article, and one case report.

The review by Dana Goldenberg, Caroline McLaughlin, Srinivas V. Koduru, and Dino J. Ravnic (8) sheds light on the current methodologies, clinical achievements, barriers, and future perspectives of regenerative engineering. The authors’ analysis underlines how regenerative engineering is a rapidly expanding interdisciplinary field that aims to provide biological substitutes for wounded tissues and organs, representing an option for those pathologies, congenital disabilities, and traumatic injuries that conventional pharmacologic or surgical interventions cannot treat. While various stem cell sources are often used as the starting platform, an engineered three-dimensional microenvironment fosters cellular differentiation into functional tissue. Tissue assembly can be facilitated by some manufacturing processes. Therefore, a multidisciplinary approach is needed to allow such science to progress. Bioengineered constructs may represent a superior model for preclinical disease and drug response modelling. The clinical translation is still on its way, especially with thin or avascular structures. As science is progressing fast, the authors stress the importance of such a topic in surgery, and the fact that surgeons should become familiar with the subject matter and its ongoing progress.

In accordance with the results of Goldenberg and colleagues, the case report by Yang Kun, Shi Mengdong, Fu Cong, and Huo Ran (9) discusses the application of tissue reengineering in the treatment of Cutis Laxa, a rare connective tissue disorder that causes the skin to become inelastic and sagging. The case reports a patient with Congenital Cutis Laxa who had plastic surgical treatment. After five months, the treatment’s results proved to represent a valid option, with no indicators of recurrence.

The review by Xingdou Mu, Juliang Zhang, and Yue Jiang (10) has a more specific focus than that of Goldenberg and colleagues, aiming to summarize studies in which 3D printing technology was used in breast reconstructive surgical procedures, understanding the future directions and applications of 3D bio-printing. The research highlights tissue engineering’s ability to develop acceptable replacements for breast-operated patients using new materials, even in complex medical conditions, including sophisticated anatomical surgical considerations or precise reconstructive operations. In addition, 3D bioprinting, which mixes cells with biomaterial scaffolds, represents a promising technology in tissue engineering.

Last but not least, the original study by Sue Yuan, Honghong Wang, and Jie Zhou (11) aims to identify and assess the risk variables for hernia in men and women with rectus abdominis diastasis (RAD). Between 2009 and 2018, the investigation included 1,294 RAD patients from six institutions within the Partners Healthcare System in Massachusetts, USA. Univariate and multivariable binary logistic regression models were used to examine risk factors. Findings reported how hernia prevalence and risk factors in women with RAD differed significantly from those in males with RAD. While umbilical hernia stands as a notable type of hernia, alcohol use and depressive disorder (mostly for male patients), and age and smoking (mostly for female patients), represent relevant risk factors.

All in all, the contributions of the research topic “Scaffold Technology, Tissue and Organ Engineering: New horizons in surgery” highlight the promising future of regenerative medicine in the surgical practice and the global clinical scenario, offering some valuable “bites” under different lenses – a comprehensive literature review, a more focused literature search in a specific clinical context, a case report regarding a rare tissue disorder, and an original study on one more common disease. The contributions of the research topic underline how, while more research is needed to reach the full clinical translation, regenerative medicine represents an exciting yet up-and-coming science for the surgical community. Therefore, scientists should be encouraged to join forces with clinical professionals and the healthcare industry in a multidisciplinary perspective to foster the progress of such a bright field of study.
